# Reliability of a Simple Physical Therapist Screening Tool to Assess Errors during Resistance Exercises for Musculoskeletal Pain

**DOI:** 10.1155/2014/961748

**Published:** 2014-03-13

**Authors:** Kenneth Jay, Emil Sundstrup, Lars L. Andersen

**Affiliations:** ^1^National Research Centre for the Working Environment, Lersø Parkalle 105, 2100 Copenhagen Ø, Denmark; ^2^Electronics and Computer Science, Faculty of Physical and Applied Sciences, University of Southampton, Southampton SO17 1BJ, UK; ^3^Institute of Sports Science and Clinical Biomechanics, University of Southern Denmark, Campusvej 55, 5230 Odense, Denmark

## Abstract

The main objective was to investigate the intra- and intertester reliability of a simple screening tool assessing errors in exercise execution by visual observation. 38 participants with no previous resistance exercise experience practiced for two weeks four typical upper limb exercises using elastic tubing. At 2-week follow-up, the participants were invited for a test-retest assessment on errors in technical execution. The assessment was based on ordinal deviation of joint position from neutral of the shoulder, elbow, and wrist in a single plane by visual observation. Moderate intratester reliability weighted kappa (w*Κ*) score ranging from 0.50 (0.21–0.71) to 0.57 (0.24–0.82) for observer 1 and a fair to moderate intratester reliability w*Κ* score ranging from 0.27 (0.09–0.43) to 0.52 (0.15–0.86) for observer 2 across the four exercises was observed. For intertester reliability moderate to substantial mean w*Κ* scores were found between the two observers, slightly improving from round one to round two ranging from 0.40 (0.20–0.59) to 0.68 (0.45–0.91) in round one to 0.52 (0.20–0.80) to 0.69 (0.39–0.86) in round two. The exercise error assessment demonstrated fair to substantial intratester and intertester reliability, which is congruent with previously published studies. Hence the simplicity of defining a neutral joint position for each of the involved joints in the exercise and categorizing the deviation in “some deviation” and “substantial deviation” to either side in a single plane is a viable and inexpensive solution when assessing for errors during exercise.

## 1. Introduction

With an estimated cost between 0.5% and 2% of the Gross National Product [1] musculoskeletal disorders comprise one of the most common and costly public health problems in Europe today [2]. In particular, the occurrence of neck and shoulder pain has been progressively increasing through the past 10–20 years [3] and is now only surpassed by low back pain, which still is the most common reported multifactorial musculoskeletal disorder [4, 5]. In occupations characterized by repetitive movement tasks and sustained low force muscular contractions, such as computer and laboratory work, neck and shoulder pain is of substantial concern [6, 7] as pain not only decreases wellbeing at work [1, 8–10] but could also affect productivity and accuracy, something of utmost importance in the work of laboratory technicians [11], while potentially leading to an increased sense of stress [12]. Indeed, the socioeconomic consequences of musculoskeletal pain and discomfort are considerable.

Possible intervention strategies for reducing the adverse effects of monotonous repetitive work include strengthening exercises of the painful muscles and a substantial number of studies within the past 10 years provide ample evidence for the effectiveness and the clinical relevance of physical activity in the form of resistance training modalities to manage musculoskeletal discomfort and pain [1, 13–16]. In accordance with this, our lab has previously shown strong effects on neck and shoulder pain with resistance exercise using elastic tubing, dumbbells and kettlebells [15, 17–25] among office workers and lab technicians with substantial and clinically relevant pain reduction. However, one possible concern with integrated resistance exercise as an intervention strategy in the working environment is the potential harmful effects of incorrectly executed exercises. Although, to own knowledge, no cause and effect studies provide evidence that wrongly executed strengthening exercises musculoskeletal problems it may be speculated that improper exercise form potentially increases the risk of sprains, strains, tendonitis, bursitis, or impingement of joints and ligaments as well as muscle contusions and general overuse injuries [26, 27]. For instance, the impingement of the subacromial bursa lying between the coracoacromial ligament and the supraspinatus muscle [28] is a relatively common problem in rehabilitation of athletes and it may occur with overuse and/or lack of scapulae-humeral rhythm during shoulder abduction movements as seen in swimmers [29–32].

To minimize mistakes potentially leading to overuse injuries, physical therapists and physical trainers may benefit from a consensus about the correct technical execution of the exercise by utilizing an assessment tool. Additionally, no standardized assessment tools exist at present, whereas the main objective of this study was to develop and investigate the intra- and intertester reliability of a simplistic assessment tool to assess errors in exercise execution to help clinicians evaluate technical errors in exercise execution. Four commonly used elastic tubing exercises for upper limb musculoskeletal pain were included.

## 2. Methods

### 2.1. Study Design

We recruited 38 participants (laboratory technicians and office workers) from a pool of approximately 200 people at a large pharmaceutical company in Copenhagen, Denmark, in the fall of 2012. Inclusion criteria were (1) a history of neck or shoulder pain, (2) female aged 18–67 years, and (3) no prior experience exercising with elastic tubing. Exclusion criteria were (1) resting blood pressure higher than 160/100, (2) pregnancy, and (3) life-threatening disease or other adverse health conditions and contraindications towards resistance exercise. The participants were recruited based on their answers to a recruitment-screening questionnaire sent out by email. Participants meeting the inclusion criteria were allocated to either a personal + video instruction group or a video-based instruction group as part of a randomized controlled trial that will be published in a separate article. Following two weeks of practicing four different elastic tubing exercises targeting the shoulder, arm, and hand musculature, all participants were invited to participate in intra- and intertester reliability examination of the assessment tool for errors made during exercise execution.

### 2.2. Ethical Approval and Trial Registration

All participants (*n* = 38) were informed about the main objective and content of the project and gave written informed consent to participate in the study, which conformed to the Declaration of Helsinki. The study was approved by the Local Ethical Committee (H-3-2010-062).  Table 1 shows baseline demographics.

### 2.3. Exercise Error Assessment

**Figure 1:**
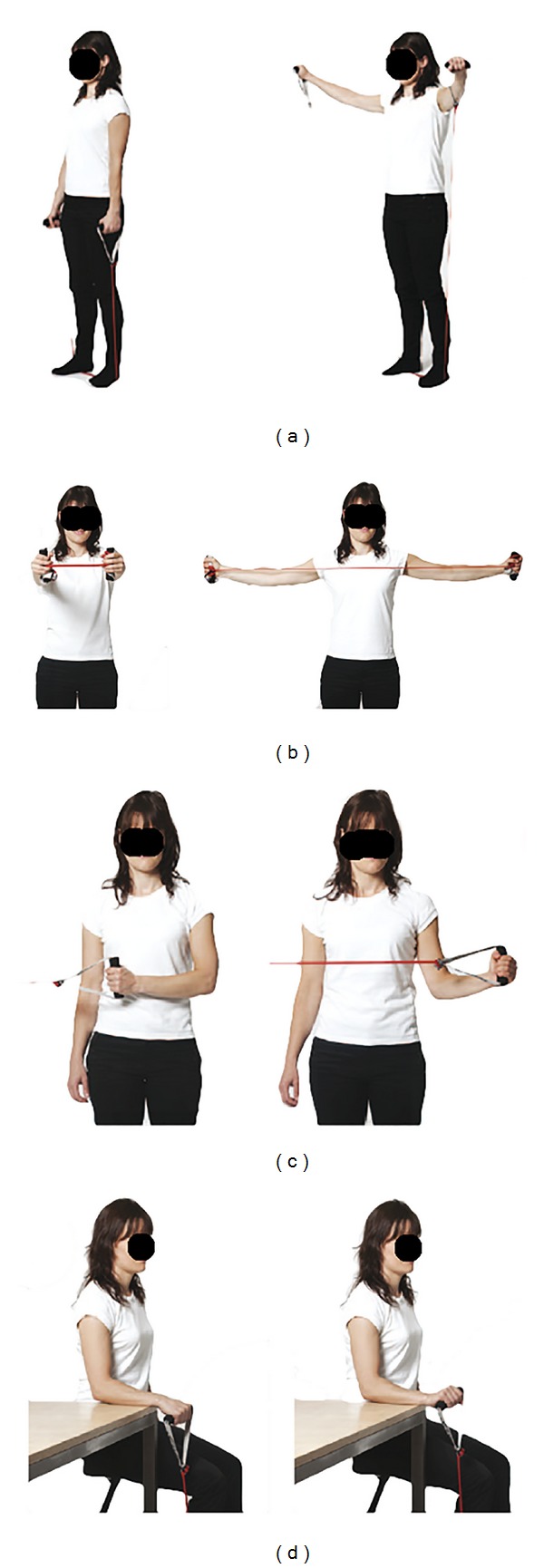
(a)–(d) show the start and finish position (ideal) of the four elastic tubing rehabilitation exercises used for examiner inter- and intratester reliability testing. Pictures from http://www.jobogkrop.dk/.

**Figure 2:**
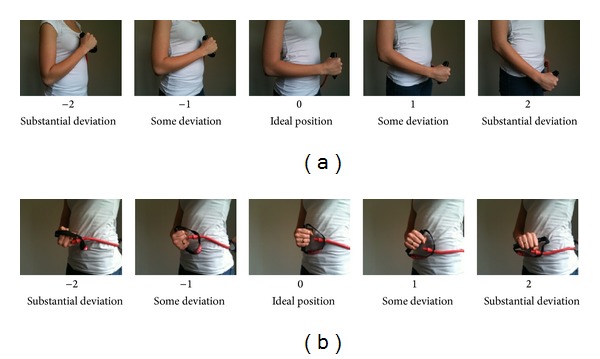
(a) and (b) provide examples of how the joint positions deviations were observed and evaluated. The picture series show possible error positions to either side from the ideal position of the elbow in the sagittal plane from a side view (a) and supination/pronation deviations from ideal of the forearm in the unilateral shoulder external rotation exercise. Pictures show the right side. Negative deviations were denoted for angles below 90 degrees of the joint and positive deviations were applied to angles above 90 degrees.

Together with two physical therapists we developed a simple standardized operating procedure for assessing the number of errors in four common shoulder, arm, and hand exercises using elastic tubing. The four exercises were (1) bilateral raise, (2) unilateral external shoulder rotation, (3) unilateral wrist extension, and (4) bilateral scapular retraction (Figures 1(a)–1(d)). Each exercise was described by joint (wrist, elbow, and shoulder) and ordinal deviation from the neutral position in a single plane, by visual observation. For each joint the examiners had to evaluate by how much the position of the joint deviated from neutral, as well as to what side from neutral, during exercise execution each joint deviation was chosen based on best practice and instructional experience with these exercises. The possible deviations were denoted as “no deviation,” “some deviation,” or “substantial deviation.” The assessment score “no deviation” was given the value “0,” the score “some deviation” was given the value either “+1” or “−1” depending on which direction the deviation had and “substantial deviation” was given the value “+2” or “−2” again depending on the direction of the deviation equaling five different possible scores (−2, −1, 0, 1, and 2) with “0” being neutral or the defined ideal for each of the exercise specific subdomains.  Figure 2 shows an example of the elbow position in the shoulder external rotation exercise for the right side.  Table 2 lists the subdomains for each exercise. Figures 1(a)–1(d) show the four exercises with the ideal defined technique (no deviation) in the start and end position and video instructional material can be seen online here: http://www.jobogkrop.dk/Ondt-i-muskler-og-led/Ondt-i-nakke-skulder-og-arm/Elastikoevelser-for-nakke-skulder-og-arm.

### 2.4. Procedure

For the reliability assessment each participant was invited in for assessment on two separate occasions with at least one day in between, by two trained physical therapists. They were asked to perform 2 × 10 repetitions of each exercise in a slow and controlled manner taking approximately 1-2 sec. for the concentric portion of the lift and 1-2 sec for the eccentric portion. For the unilateral exercises (unilateral shoulder external rotation and unilateral wrist extension) the participant used the dominant arm. One set of each exercise was demonstrated facing the examiners and one set was demonstrated in a side-view profile. The two examiners conducting the assessment were positioned in the room in such a way that they could not see what the other examiner was noting. The examiners were instructed to not talk about the exercise execution during or after the assessment. Furthermore, the examiners were instructed to not provide any feedback to the participant on the execution of each exercise. Finally a standard operating procedure was followed to make sure each assessor evaluated the appropriate technical aspects of each exercise.

### 2.5. Dropouts

Approximately 200 people received information email about the study. 49 people agreed to answer a baseline-screening questionnaire and 38 were invited to participate in the study. One person was excluded due to lack of answering the screening questionnaire and four people did not show up for the second assessment by the examiners due to sickness unrelated to the study. Thus, 37 people completed the first assessment and 33 people completed the second assessment.

### 2.6. Statistics

Dropouts from the two-week familiarization training were invited to participate in the test-retest assessment to avoid selection bias. Intra- and intertester reliabilities were determined by weighted Kappa (w*Κ*) analysis of the SAS statistical software (SAS institute, Cary, NC, version 9.2). Landis and Koch have previously defined w*Κ* > 0.80 as almost perfect, 0.60 ≤ w*Κ* < 0.80 as substantial, 0.40 ≤ w*Κ* < 0.60 as moderate, 0.21 ≤ w*Κ* < 0.40 as fair, and w*Κ* < 0.20 as slight agreement [33]. Further, we calculated an intraclass correlation coefficient (ICC) between the two examiners and two rounds from the mean error assessment scores of each exercise.

## 3. Results

In general, we found a moderate intratester reliability mean w*Κ* score ranging from 0.50 (0.21–0.71) to 0.57 (0.24–0.82) for observer 1 and a fair to moderate intratester reliability mean w*Κ* score ranging from 0.27 (0.09–0.43) to 0.52 (0.15–0.86) for observer 2 across the four exercises. Similarly, a moderate to substantial intertester reliability mean w*Κ* was found between the two observers, slightly improving from round one to round two ranging from 0.40 (0.20–0.59) to 0.68 (0.45–0.91) in round one to 0.52 (0.20–0.80) to 0.69 (0.39–0.86) in round two.  Table 2 summarizes the intra- and intertester reliability w*Κ* scores in each of the observed subdomains of the four different exercises (Figures 1(a)–1(d)) and Table 3 summarizes ICC for the four exercises.

## 4. Discussion

This study shows fair to substantial intra- and intertester reliability of a very simple design assessment protocol of errors performed during commonly used elastic tubing exercises for musculoskeletal pain of the neck/shoulder, arm, and hand. The results show that physical therapists and physical trainers, with little practice, are able to spot errors in trainee exercise execution in a reliable way once a consensus about correct technical execution has been formed. Our results compliment previous findings in intertester reliability of movement assessments with similar results. For instance, the Melbourne Assessment of Unilateral Upper Limb Function for children with neurological impairments was found to have moderate to high interrater reliability by visual observation [34] and similarly; the Movement Assessment Battery for Chinese preschool children (Movement ABC) has also shown good intertester reliability [35]. Movement assessments targeted at the healthy adult population, like the Functional Movement Screen (FMS), have shown equally good intratester and intertester reliability [32, 36, 37] indicating that assessing movement by visual observation, between testers as well for the same tester, is a usable tool when physical trainers, physical therapists, and movement coaches correct technical exercise execution.

Overall our study shows a moderate reliability of the assessment tool. Noteworthy is the increase in intertester reliability from round 1 to round 2 in three of the four exercises indicating that examiners increase their accuracy in error spotting, which can be construed as a basic visual discrimination task. That kind of perceptual learning has been shown to improve with practice and can be viewed as local (in a retinotopic sense), as well as specific to the orientation of the visual target [38, 39], arguably the case in our study.

The one exercise not showing an improvement in intertester reliability between rounds is the bilateral scapulae retraction exercise. Investigating the subdomains indicates that the wrist and elbow position assessment decreases between the two examiners, that is, intertester reliability, from round 1 to round 2. It could be speculated that, because the majority of movement happens around the shoulder joint in the Bilateral raise, examiners might unintentionally pay more attention to that, because of the dynamic movement happening in that joint (shoulder) compared to the smaller joints (wrist and elbow), which primarily hold a static position throughout the movement. Directing attention towards the major moving part of the body could be an indication of momentary attentional drift of the examiner [40], but it still remains unclear why there is a drop in w*Κ* intertester reliability score in this particular exercise from round 1 to round 2. The intratester reliability of the two examiners in this study was fair to moderate with observer two showing poorer reproducibility of the assessment, especially when assessing unilateral shoulder external rotation. The lack of consistency between rounds for examiner 2 is difficult, if not impossible, to explain but again may be related to an attentional drift of the mind resulting in momentary inattentiveness.

Strengths of the present study include the number of participants being assessed and the simple assessment design. A further strength is that we assessed both intra- and intertester reliabilities, which gives information about reproducibility over time as well as between different assessors. Limitations to the present study include the lack of objective assessment measures, for example, joint angle kinematics and the constrained exercises demonstration of each participant limited to 2 sets of 10 repetitions. Had the exercise performance of each participant been recorded and the examiners allowed to see the video multiple times as well as having the option of slowing and freeze framing the video sequences the reliability scores might have been higher. Furthermore, having video footage of the examiners performing the assessments would have allowed our lab to analyze behaviour and state of attention. However, in most settings where time and equipment are limited such options are not viable, and simple screening tools are needed. Finally, it could be argued that the study is limited by only testing the reliability between two assessors. Theoretically, these reviewers could have been of above average visual assessment ability, which could provide a skewed result of inter- and intrarater reliability.

In conclusion the exercise error assessment demonstrated fair to substantial intratester and intertester reliability, which is congruent with previously published studies on movement assessment reliability, hence the simplicity of defining a neutral joint position for each of the involved joints in the exercise and categorizing the deviation in “some deviation” and “substantial deviation” to either side in a single plane is a viable solution when assessing simple exercises for errors during execution.

## Figures and Tables

**Table 1:** Shows baseline demographics before group allocation.

	Baseline demographics	
	Mean (SD)	Range
Number of participants	38	N/A
Age (years)	45 (8.1)	24–59
Weight (kg)	68.2 (6.9)	55–85
Height (cm)	169.9 (9.5)	156–187

**Table:** (a) Exercise: Bilateral raise

Joint	Ordinal deviation	Left (L)/Right (R)	Intratester reliability		Intertester reliability	
			Observer 1	Observer 2	Round 1	Round 2
Wrist	Palmar/dorsal flexion	L	0,39 (0,10–0,68)	0,31 (0,01–0,60)	0,52 (0,31–0,72)	0,33 (0,10–0,55)
		R	0,57 (0,34–0,79)	0,52 (0,25–0,79)	0,36 (0,17–0,55)	0,44 (0,25–0,63)
	Radial/ulnar deviation	L	0,46 (0,07–0,85)	0,43 (−0,02–0,89)	0,30 (−0,05–0,66)	0,84 (0,53–1)
		R	0,37 (−0,06–0,79)	0,20 (−0,27–0,67)	0,22 (−0,14–0,59)	1 (1-1)

Elbow	Flexion/extension	L	0,25 (−0,18–0,68)	0,38 (−0,15–0,90)	0,51 (0,14–0,88)	0,49 (−0,09–1)
		R	−0,04 (−0,10–0,03)	0,31 (−0,17–0,78)	0,41 (0.00–0,83)	0,38 (−0,15–0,91)

Shoulder	Transverse plane position	L	0,43 (0,12–0,75)	0,37 (0,07–0,67)	0,58 (0,32–0,83)	0,38 (0,09–0,66)
		R	0,38 (0,09–0,67)	0,30 (0,01–0,59)	0,55 (0,29–0,8)	0,40 (0,12–0,67)
	Abduction/adduction	L	0,72 (0,46–0,97)	0,46 (0,20–0,71)	0,40 (0,18–0,62)	0,59 (0,31–0,88)
		R	0,61 (0,32–0,89)	0,41 (0,15–0,68)	0,42 (0,20–0,64)	0,56 (0,28–0,85)
	Internal/external rotation	L	0,76 (0,50–1.00)	1.00 (1.00-1.00)	0,16 (−0,12–0,44)	0,15 (−0,12–0,42)
		R	0,76 (0,51–1)	1 (1-1)	0,16 (−0,12–0,44)	0,15 (−0,12–0,43)

		Mean	**0,55 (0,30**–**0,79)**	**0,50 (0,16**–**0,81)**	**0,42 (0,09**–**0,71)**	**0,55 (0,30**–**0,79)**

**Table:** (b) Exercise: Bilateral shoulder retraction

Joint	Ordinal deviation	Left (L)/Right (R)	Intratester reliability		Intertester reliability	
			Observer 1	Observer 2	Round 1	Round 2
Wrist	Radial/ulnar deviation	L	0,64 (0,18–1)	0,54 (0,08–1)	0,55 (0,17–0,93)	0,37 (−0,18–0,92)
		R	0,64 (0,18–1)	0,54 (0,08–1)	0,39 (−0,03–0,81)	0,37 (−0,18–0,92)
	Palmar/dorsal flexion	L	0,53 (0,31–0,76)	0,34 (0,10–0,57)	0,60 (0,43–0,78)	0,60 (0,40–0,79)
		R	0,52 (0,31–0,74)	0,39 (0,14–0,64)	0,61 (0,44–0,78)	0,50 (0,31–0,70)

Elbow	Flexion/extension	L	0,62 (0,24–1)	0,37 (−0,16–0,90)	0,69 (0,36–1)	0,48 (−0,12–1)
		R	0,62 (0,24–1)	0,37 (−0,16–0,90)	0,69 (0,38–1)	0,48 (−0,12–1)

Shoulder	Transverse plane position	L	0,47 (0,27–0,67)	0,41 (0,19–0,63)	0,46 (0,22–0,71)	0,73 (0,56–0,90)
		R	0,47 (0,27–0,67)	0,41 (0,19–0,63)	0,46 (0,22–0,71)	0,73 (0,56–0,90)
	Internal/external rotation	L	0,65 (0,32–0,97)	0,84 (0,61–1)	0,77 (0,55–0,98)	0,85 (0,64–1)
		R	0,65 (0,33–0,97)	0,84 (0,61–1)	0,92 (0,77–1)	0,74 (0,48–1)
	Abduction	L	0,68 (0,36–1)	0,45 (0,01–0,89)	0,70 (0,37–1)	0,72 (0,35–1)
		R	0,68 (0,36–1)	0,45 (0,01–0,89)	0,70 (0,37–1)	0,72 (0,35–1)

		Mean	**0,51 (0,22**–**0,78)**	**0,52 (0,15**–**0,86)**	**0,68 (0,45**–**0,91)**	**0,52 (0,20**–**0,80)**

**Table:** (c) Exercise: Unilateral shoulder external rotation

Joint	Ordinal deviation	Left (L)/Right (R)	Intratester reliability		Intertester reliability	
			Observer 1	Observer 2	Round 1	Round 2
Wrist	Palmar/dorsal flexion	R	0,35 (0,07–0,63)	0,58 (0,35–0,82)	0,56 (0,33–0,78)	0,76 (0,58–0,94)
	Radial/ulnar deviation	R	0 (0-0)	0 (0-0)	0,79 (0,38–1)	1 (0,62–1)

Elbow	Supination/pronation	R	1 (0,50–1)	0 (0-0)	0 (0-0)	1 (0,50–1)
	Flexion/extension	R	0,52 (0,27–0,76)	0,14 (−0,19–0,46)	0,36 (0,13–0,59)	0,41 (0,17–0,65)

Shoulder	Abduction	R	0,52 (0,27–0,77)	−0,04 (−0,10–0,03)	0,34 (0,09–0,60)	0,49 (0,21–0,77)
	Flexion/Extension	R	0,40 (−0,02–0,83)	0,14 (−0,21–0,48)	0,31 (−0,08–0,70)	0,50 (0,13–0,88)

		Mean	**0,50 (0,21**–**0,71)**	**0,27 (0,09**–**0,43)**	**0,52 (0,29**–**0,72)**	**0,69 (0,39**–**0,86)**

**Table:** (d) Exercise: Unilateral Wrist Extension

Joint	Ordinal deviation	Left (L)/Right (R)	Intratester reliability		Intertester reliability	
			Observer 1	Observer 2	Round 1	Round 2
Wrist	Palmar flexion at the bottom	R	0,67 (0,40–0,93)	0,62 (0,36–0,89)	0,79 (0,59–0,99)	0,75 (0,51–0,99)
	Dorsal flexion at the top	R	0,67 (0,33–1)	0,46 (0,10–0,82)	0,59 (0,27–0,91)	0,59 (0,23–0,95)
	Radial/ulnar deviation	R	0,37 (−0,16–0,90)	1 (0,62–1)	0 (0-0)	0 (0-0)

		Mean	**0,57 (0,24**–**0,82)**	**0,44 (0,20**–**0,60)**	**0,40 (0,20**–**0,59)**	**0,62 (0,34**–**0,79)**

**Table 3:** Summarizes the intraclass correlation coefficient between testers and rounds.

	Intraclass correlation coefficient (ICC)			
	Examiner 1	Examiner 2	Round 1	Round 2
	Round 1-2	Round 1-2	Examiner 1-2	Examiner 1-2
Bilateral raise	0.47	0.77	0.68	0.49
Bilateral scapular retraction	0.77	0.71	0.82	0.82
Unilateral shoulder external rotation	0.68	0.26	0.67	0.83
Unilateral wrist extension	0.75	0.61	0.81	0.77
